# Supplementary material for: Sub pixel resolution using spectral-spatial encoding in x-ray imaging
Source: PLoS One. 2021 Oct 27;16(10):e0258481. doi: 10.1371/journal.pone.0258481 (PMC8550400; doi:10.1371/journal.pone.0258481)
Supplement: Attachment — Submitted filename: author_response_letter_PONE_final.docx [file pone.0258481.s001.docx]

PONE-S-21-15596

Title: Sub pixel resolution using spectral-spatial encoding in x-ray imaging

Response letter to associated editor and reviewer comments

We will respond to the associate editor, and then the reviewer in separate tables below.

We made changes to the manuscript, all marked in red text, along with modification of some figures per reviewer requests.

At a high level, this paper introduces an entirely new method for super resolution. We explain the basis for the method via presenting the mathematics describing the measurement process for multiple means of realizing SPE. The numerical simulations are presented not to prove in some rigorous way SPE works, but merely to demonstrate for those problem conditions SPE works. We’ve added text to make it clear we demonstrate SPE via presenting its mathematical foundation and show it is feasible with simulations. Full characterization of SPE is of course something we want to do for specific systems, at which time using metrics like MTF, NPS, detectability, dose efficiency etc. would be used. This is the foundation paper for the method, it is not meant to both introduce a new imaging acquisition technique and fully characterize it for some specific application. **This is why we went to plos one.**  We like this journal, instead of a medical physics or industrial imaging journal, this journal sits between multiple disciplines, the perfect place to introduce a new imaging technique. But in our opinion, not the ideal place to characterize using a problem instance and metrics specific to some niche subset of the readers.

Please don’t judge our paper on the simulations alone, the methods should stand on the mathematical explanation of SPE with the simulations providing examples it is feasible. Reviewers could ask for more and more regarding simulations until the paper is consumed by methods for numerical simulations and the metrics used to analyze them. Such a paper has a place in the literature, after this foundational paper for the SPE method is published. **We appeal to the AE and reviewers to understand this.** No negative reviewer comments mentioned the mathematical foundation we lay out in this paper.

AE comments

| Thank you for submitting your manuscript to PLOS ONE. After careful consideration, we feel the manuscript presents a new and potentially interesting method for improving spatial resolution, but the current presentation of data does not support the conclusion that spatial resolution was improved. Therefore, we invite you to submit a revised version of the manuscript that addresses the points raised by each reviewer.  Most critically, both reviewers found support for the conclusion of sub-pixel resolution to be weak or questionable, due to a lack of quantitative assessment and limitations in the simulations.  Reviewer 1 also requested a more quantitative assessment of image noise. Both reviewers also highlighted that results for a photon-counting detector would provide greater support for the conclusion.  Both reviewers suggested a number helpful clarifications and improvements in the manuscript presentation. | Thanks for taking the time to handle our article. Our paper’s method is demonstrated in mathematical explanation, and we gave some examples using numerical simulation. We stressed that there are a huge number of free parameters, and almost as many metrics one could use to assess our method. This paper introduces the concept of SPE. If the reviewers and AE have issue with the mathematical explanation of SPE or in the specific examples we show, we can address them. But adding new metrics to our example simulation and or new simulations for different data acquisitions is not something we desire to do.  There were some fundamental mis-understandings with our approach by the review team (reviewer 2 commented that we need 2 readouts, one for each side of the detector…the entire method of SPE is to mitigate the need for this), we will hopefully be able to mitigate those mis-understandings in this letter. |
| --- | --- |
| The editor's assessment is that the author's deserve an opportunity to overcome these major concerns, but it may not be possible to convincingly demonstrate sub-pixel resolution rather than merely improvements in image contrast due to basis material decomposition, which is already accomplished in dual-energy CT. | The gains we observed in resolution, by seeing the bone high frequency pattern and the air defects do not rely on contrast improvements. Those are high contrast imaging tasks, enabled only by our increase of spatial resolution via SPE. For example, we show monochromatic profiles from 1 mm, 2 mm, and 1 mm SPE derived results. The pattern is only observable at the true 1 mm detector and the 1 mm SPE derived detector, demonstrating our visualization of the high frequency bone pattern and the air defects had not to do with DECT derived contrast enhancements, but an increase in spatial resolution. |

Reviewer 1

| The authors in this manuscript describe a spatial-spectral sub-pixel encoding (SPE) scheme to achieve super resolution. The idea is interesting, and as demonstrated by the authors, has clear benefits, specifically for industrial applications where radiation dose is not a concern. I have the following suggestions to the authors. | Thank you for taking the time to review this article for us! And thanks for the kind words on our method. |
| --- | --- |
| The points about noise amplification appear to be qualitatively described. The authors should consider describing the noise amplification objectively, using measurements from the original 2 mm image, 1 mm image and 2 mm SPE image. This will give the readers an idea as to how strong the noise (amplification) is, to emphasize the importance of noise reduction strategies. | We do only qualitatively describe them.  But we include an analysis on covariance matrix. This is far more important for someone trying to understand SPE than the absolution noise values. Covariance matrix will let someone understand the fundamental relationship of noise correlations, of which SPE has correlations. For this introduction paper to SPE, quantifying noise amplification is not fair, since we haven’t optimized the simulations. The simulations serve only to demonstrate SPE is feasible. The covariance matrix analysis shows a high level facet of SPE noise performance, mainly that correlations exist and what their polarity is.  We describe in the paper how SPE is non-linear, and noise amplification will depend on specific problem instances. We don’t want to spend a lot of time and space in this first paper characterizes the specific simulations we chose to demonstrate SPE. The support/explanation/demonstration of SPE is in the math, and these simulations simply demonstrate it is feasible for the conditions we simulated. Quantifying noise for these example simulations would not be fair to SPE, SPE needs to be optimized for a specific problem instance (i.e., imaging task) before it should be characterized using a metric like noise. For all we know, there are problem instances where noise reduction algorithms are not needed. Or for our industrial example, maybe that amount of noise is fine…we do enable the defect to be seen using a realistic exposure level one would use today on an industrial imaging system.  We don’t mean to just “explain away” the need to do more here, but this is the first paper defining an entire new method for super resolution. We hope it can rest on its mathematical foundation, and the simulations are present to convince people it worked for these conditions. |
| There is a description for realizing SPE using photon-counting detectors (2.3), however the simulations and results are for energy-integrating detector. Can the authors provide a simple example simulation for a photon-counting detector setup? This could strengthen the manuscript. | This paper focuses on energy integrating detectors because they are, by far, the most common detector type used in industrial and medical today. We understand the benefits of photon counting detectors and will consider them in future, application specific work. |
| Page 7, Lines 114 to 116: "Here we have allowed the incident spectra and the detector response to vary for the left and right sides of the detector pixel. We have also accounted for the presence of the filter shown in Figure 1 using F(E) which takes on different values for the left and right sides of the detector." I have trouble understanding this statement. If I understood the SPE correctly, the detector response for left and right sides of the pixel would be identical, considering that the actual pixel (with two defined sub-pixel regions) is a single unit, therefore has a single response instead of two? The variation in response is introduced in F(E) due to the filters, if this is what the authors are referring to. Please rephrase and clarify. | We changed this to make it clearer. The detector could be made such that it has a different type of semiconductor or scintillator on the let and right sides, albeit it still only has a single read out.  Changed to this now  “Here we have allowed the incident spectra and the detector response to vary for the left and right sides of the detector pixel while keeping the detector element with a single read out.  The detector response could vary by using a different scintillator or semiconductor material on the left and right sides of individual detector elements (i.e., the element would still have a single readout, but regions of varying sensitivity to x-rays that would vary with energy). We have also accounted for the presence of the filter shown in Figure \ref{fig1} using $F(E)$ which takes on different values for the left and right sides of the detector. |
| Figure 2: For a general reader, it is hard to locate the 4 mm bone pattern referred by the authors. I suggest adding arrow marks to point to the regions of interest, and possibly include zoomed-in inset images. Also, it would help to include a schematic of the phantom design. | Thanks, this is a good observations, we changed this by editing the figure and caption. |
| Figure 4: Window setting is [0.4 0.5], please state the unit for the image intensity value. | Those figures have no units. They are not a.u. either. They are line integral arguments to an exponential function, so they have no unit. See the methods sections where we describe how we calculate those images.  We added a note to the figure caption explaining these images are the arguments to an exponential function to help readers not familiar with x-ray imaging. |
| The line profiles in Figure 3 are a semi-quantitative way to show spatial resolution improvements. I request the authors to consider reporting MTF if possible, using the slanted-edge technique (based on edge spread function). This is a standard method widely accepted in the imaging community to quantify spatial resolution for new systems or techniques. This is another addition that I feel can strengthen this manuscript, and request the authors to consider this metric. | To make this manuscript accessible to both medical and industrial CT users, we choose simple but obvious indicators of the increased resolution afforded by SPE. As an example of the difference between Industrial and medical use cases: industrial only provides guidance for CT MTF and uses a step edge of a metal disk (ASTM E1695-20).  We agree imaging of a slanted edge is a powerful metric for demonstrating spatial resolution gains. We plan to include this and other metrics in future work dedicated to fully characterizing the performance of SPE for specific applications. |

Reviewer 2

| This is a review of "Sub pixel resolution using spectral-spacial encoding in x-ray imaging". The authors present a new technique named sub pixel encoding (SPE) to achieve super resolution in imaging. The authors present arguments where this technique is superior to previous methods where the object and/or imaging system components do not have to be physically moved. | Thanks for taking the time to review. We agree with this summary of our work. |
| --- | --- |
| The major concern with the manuscript is the authors have not convincingly demonstrated that spatial resolution has been improved. Rather, the approach and data would support a new method for basis material decomposition which is more likely enabling the ability to resolving objects. While this work was demonstrated through simulations, it is not apparent this would actually work on an imaging system with energy integrating detectors where there wouldn't be separate readouts for the described left and right filters. The authors do not address this but weighting factors could be used as an estimate which is done in some dual-energy CT applications. However, this would require a prior knowledge and potentially limit the application in medical imaging. | We do not require separate read outs for the left and right sides of the detectors. This is in fact the strength of the SPE technique. See figure 1, in one implementation of SPE we have filters over different regions of the same detector element 🡪 2 filtered regions with 1 read out. The primary focus of this paper is the novel method for spatial resolution enhancement, but you are correct that DECT is a second benefit for this approach.  Regarding DECT via filtering different regions of the detector, this is a well-known clinical product. Siemens makes a product called “Twin Beam” where they have a gold and tin filter on different regions of the bowtie filter. We are not purposing to do this here. |
| 1. There isn't a discussion on the choice of filter material along with potential other materials that could be considered. | This is true. In the first draft, we just say that future work is needed to look at optimization of filters. We added a new section in the manuscript which discusses what happens when one changes filter material and relative thicknesses.  Where we previously just said this “As the F filter becomes so thick as to block the the detector signal to part of the detector completely, SPE approaches a ``high resolution comb'' like spatial resolution increasing solution” and then we cite the medical imaging approach from Siemens where they do this.  We added new text to the manuscript to add to this  “Preliminary studies by our group have shown that as the filter material is changed to higher atomic number or made thicker on one side versus the other, stronger changes in filter value are obtained with energy. This is good for providing the independent measurements required by SPE on the different regions of the detector. Such conditions, however, increase the attenuation of the signal if the filter is placed between the object and the detector read out which increases noise. So careful optimization of the filter material and thickness and placement is required in future studies of the SPE method.” |
| 2. It is not clear in the methods if the fluence rate was adjusted when kV changes were applied. | We added text to make it clear that we used spectra generators which inherently change fluence rate when the spectra is changed.  We added this text to both simulation sections “The spectrum generator uses modeled and measured data to provide realistic X-ray spectra for the simulated techniques and inherent filtrations.” |
| 3. It is not clear in the methods how the detector response was modeled for different energies. | In the methods we stated the detector was modelled with a linear energy response and a perfect detection efficiency. Yes, the response is not exactly linear and yes, the detection efficiency will not be perfect, but these assumptions are routinely made in the medical physics literature. For proof of concept, we don’t think a more complicated detector model adds significant value. |
| 4. Figure 2 would be improved if an image of the digital reference object was presented. It is difficult to appreciate what was being imaged. | Thanks, we have modified this figure to include a digital reference object showing how the phantom is composed. |
| 5. The ability to resolve the defects using SPE in Figure 4(c) is not very convincing. An observer would have to have more information for interpretation. | We are not sure what additional information the reviewer is referring to. These images along with the line profiles in Figure 5 clearly show only the 1 mm and 1 mm SPE derived data show the defects as separate. That is the extent of what we would like a reader to come away with. In other words, we created a phantom with a defect requiring 1 mm spatial resolution to visualize it, and we used a 2 mm detector along with the SPE technique and we were able to resolve it.  We added text to the figure captions to make this clearer. |
| 6. The discussion on color filter arrays and demosaicking processing is confusion. Color filter arrays allow selected visible light of a particular energy and corresponding frequency to transmit. It is not clear if the point is higher frequency photons are transmitted to provide higher spatial resolution. This would likely only be applicable to high contrast objects and would degrade image contrast, thus severely limiting application. | The only point we are making here is that SPE is “in some ways” similar to color filter arrays in that both SPE and CFA encode information on the detector with filters and use this encoding to obtain information the original detector could not obtain. We are not saying SPE is analogous to CFA in all ways. Our point is not to relate frequency of x-ray photons to what happens in CFA. This section of the paper is relating SPE to other techniques. SPE was invented by us, so the literature doesn’t have SPE, so in this section we discuss a few approaches that perform similar but not completely analogous processing steps. |
| The theoretical basis and statistical approach for this work is reasonable. The data and results simply don't support the conclusions. | No reviewers or AE commented on our mathematical foundation we present for SPE. All negative criticisms revolved around the example simulations we presented. We agree that if we just showed figure 1, and then the numerical simulations, we would have a weak paper. Our current paper provides a rigorous explanation of SPE for a multitude of measurement systems (energy integrating, photon counting, filter inside detector material, filters in source, filters on top of detector, etc.) and then provides a few numerical examples to illustrate SPE is feasible.  Please don’t judge our paper on the simulations alone, the methods should stand on the mathematical explanation of SPE with the simulations providing examples it is feasible. |
