# Supplementary material for: Sub pixel resolution using spectral-spatial encoding in x-ray imaging
Source: PLoS One. 2021 Oct 27;16(10):e0258481. doi: 10.1371/journal.pone.0258481 (PMC8550400; doi:10.1371/journal.pone.0258481)
Supplement: Attachment — Submitted filename: ResponseToReviewersR2_Draft1_9_21_2021.docx [file pone.0258481.s002.docx]

Response to reviewers for the paper titled “Sub pixel resolution using spectral-spatial encoding in x-ray

imaging”

Thanks AE and reviewers! Thank you for giving us a chance to make some improvements on the last revision, and to make additional revisions this round. Looking back to our first version, we have noted many solid improvements motivated by your input, so thanks!

Associate Editor

1) Comment:
   Specifically, in comments directed to the editor, Reviewer 1 agreed that MTF and advanced quantitative metrics for image quality are too big an ask for this foundational paper, but maintains that frequent use of "noise amplification" throughout the manuscript begs for some kind of quantitative support. Reviewer 1 requests a "simple" ROI-based quantitative description of noise amplification be provided, which can include acknowledging the current suboptimal simulation parameters. Reviewer 1 further suggested the authors provide a brief supporting discussion about optimizing simulations for future work to include advanced metrics such as MTF, NPS, detectability etc.

   Response:
   We have included an roi-based comparison of noise in a uniform phantom region. We compare images generated by: 1 mm detectors and the SPE method that used only 2 mm detector data to generate 1 mm detector resolution. We’ve done this for uniform backgrounds representative of both the medical imaging and industrial simulations.

   We’ve additionally added a paragraph to the discussion section addressing how future work will include optimization utilizing advanced metrics (model observers, NPS, MTF) as requested.

We have also included a description of how the co-variance matrix can be used to estimate SNR, and report the SNR values of the cases we used in the paper.

2) Comment:
   Similarly, Reviewer 3 requested that issues surrounding the presented "covariance" should be presented along with their implications. Reviewer 3 provided a number of suggestions in this regard but communicated that his/her intent is not to demand a precise statistical analysis but necessary information to support the results and acknowledge unspoken issues with the current treatment.

   Response:
   We agree the presentation of the covariance matrix was brief and a bit confusing. We have fleshed the discussion of the covariance matrix out in the results section and revised the methods section to clarify how the covariance matrix was estimated. We discuss how one can use the matrix entries to get an “SNR” like metric for a given experimental case.

Reviewer 1:

It is our understanding reviewer 1 only provided comments directly to the associate editor

We thank this reviewer for their service to the journal, and our field!

Reviewer 3:

1) Comment:
   This manuscript presents a framework for increasing effective detector resolution via spatially varying filtering for an encoding component in the forward model of X-ray CT systems. It is primarily a proposal for a family of techniques to match the dimension of the measurements from augmented detectors to the unknown line integrals of basis materials. The approach is creative and the simulated, preliminary results are encouraging. There are improvements that would strengthen the paper, and this will be worthy of publication with what I would term minor revisions.

   Response:
   We greatly appreciate the reviewer’s assessment of our manuscript and hope the changes in this revision address their concerns.
2) Comment:
   Section 2.6 on Covariance Analysis should be more fully explained. Given the form of the forward model, the Jacobian would appear to be a function of the unknown parameters and nontrivial to compute. Was the Jacobian made available by the Matlab function cited? I’d like to see that parameterized matrix for the 4x4 example, but at the least we should know how it’s computed and plugged into (8) for the given case.

   Response:
   We agree this section was difficult to understand as it was previously presented. We hope the revised version is clearer. Briefly, for the medical case, we consider the forward model

$$s_{i}\left( PAL,PAR,PBL,PBR \right)=\int_{0}^{E_{max}} dE \Omega_{i}\left( E \right)\omega\left( E \right)\left[ F^{L}\left( E \right)e^{-c_{A}\left( E \right)PAL-c_{B}\left( E \right)PBL}+F^{R}\left( E \right)e^{-c_{A}\left( E \right)PAR-c_{B}\left( E \right)PBR} \right]$$

We take 4 measurements with 4 different kVp X-ray beams (i.e., varying the function $\Omega_{i}$ for each measurement). We thus have a mapping $f:\mathfrak{R}^{4}\to\mathfrak{R}^{4}$ with

$$\vec{s_{0}}=f\left( PAL, PAR, PBL, PBR \right)$$

that models our system in the absence of noise. The function $f$ is fully specified and we can calculate it’s Jacobian $J_{f}$. We don’t present the matrix explicitly (i.e., it is huge), but it is easily obtained by taking the 16 partial derivatives of $f$. By the inverse function theorem, under some differentiability assumptions on $f$ and $f^{-1}$, we have

$$J_{f^{-1}}\left( \vec{s_{0}} \right)=\left( J_{f}\left( PAL, PAR, PBL, PBR \right) \right)^{-1}$$

So, given some operating point $(PAL, PAR, PBL, PBR)$, we can calculate the Jacobian of the inverse by simply inverting the Jacobian of the forward model (we obviously know $PAL, \ldots, PBR$ in all our simulations so this can be done).

   If we treat our measurements as random variables, say, arising from zero mean additive noise (a reasonable approximation for energy integrating detectors when we measure a large number of photons)

$$\vec{s}=\vec{s_{0}}+\vec{n}$$

The first order approximation of the covariance matrix of $\vec{p}=f^{-1}(\vec{s})$ is given by

$$K_{p}\approx J_{f^{-1}}\left( \vec{s_{0}} \right)K_{n}J_{f^{-1}}\left( \vec{s_{0}} \right)^{T}$$

which, as mentioned above, can be calculated from $J_{f}$.

The same line of reasoning applies to the industrial case, with $f$ instead being a mapping from $\mathfrak{R}^{2}\to\mathfrak{R}^{2}$.

3) Comment:

Also, given the nonlinearity of the relationships, (8) should be given as an approximation rather than equality.

Response:
Thank you for catching this! Done.

4) Comment:
   Probably the best we could do here would be an inequality relative to the Cramer-Rao lower bound, which would require an expectation over the data space for each Jacobian entry.

Response:
We are by no means statisticians, but as a point of clarification, to calculate the Cramer-Rao lower bound, we believe we would need the Fisher information matrix. This would require the expectation of the Hessian of the log-likelihood function (or the outer product of it’s Jacobian with itself). This is not the same as the Jacobians of the forward and inverse models, which are the Jacobians we discuss in the paper, and the Jacobians to which we believe the reviewer is referring. There is also a Jacobian of the expectation value of the estimator in the Cramer-Rao lower bound for a biased estimator, but the differentiation is with respect to the unknown parameters (i.e., PAL, PAR,…) and doesn’t correspond to either of the Jacobians discussed in the paper.

We believe the most common usage of the term Cramer-Rao lower bound refers to an expression for a lower bound on the covariance of all unbiased estimators. We chose not to pursue this route because, due to the nonlinearity of our estimator, even under assumptions of additive, zero-mean Gaussian noise, we have no reason to believe our estimator is unbiased.

There is another form of the Cramer-Rao lower bound that applies to all estimators of a given bias (i.e. all estimators with the same expectation value at a given operating point [PAL, PAR, PBL, PBR]). We chose not to pursue this route for two reasons:
(1) The first order approximation of covariance we present is simpler and easier to understand
(2) Cramer-Rao provides only a lower bound on the covariance of our estimator, and could therefore be overly optimistic. We acknowledge our current first order estimate could, being an estimate, also be overly optimistic, but we have no reason to believe the Cramer-Rao lower bound would be more conservative.
(3) We don’t see much added value in lower-bounding the covariance of all estimators with the same bias as our current estimator
(4) Our current estimator is only meant for proof of concept. We plan to employ regulization strategies (e.g., total variation) to help deal with noise, so we feel devoting such a deep analysis to the current estimator’s noise properties would be unwarranted.

5) Comment:
   Especially since there would be a number of ways to increase the dimension

of the measurement space, we should also consider the case in which the

Jacobian is not a square matrix.

Response:
We agree this is an interesting direction. One could theoretically take more measurements, creating an over-determined system which could then be solved via an optimization-based approach (e.g., non-linear least squares). We believe this is beyond the scope of the current “proof-of-concept” paper, but have added a sentence to the discussion mentioning this idea.

6) Comment:
   The covariances are presented with little comment beyond noting the several negative entries. The 4x4 matrix has a relatively large (>10^4) condition number for a small matrix, raising questions about the information, with regard to the parameters, available in the 4 measurements. The results in Figures 3-6 are encouraging, but some discussion of their potential sensitivity to modeling errors and noise should appear in light of the eigenstructure of the given, approximate covariance. We must keep in mind as well that mean-squared error in an estimator includes both variance and bias^2. Further, the ill effects of variances depend on the signal strength (~mean) of the true values. In the example used, the approximated variances are high for elements 1 and 2 of the parameter vector, but I believe these are both associated with the water component, which has a much higher mean value than the bone in the case shown.

   Response:
   We agree the paper needs more commentary regarding the covariance matrix. We have added a paragraph to the results section providing interpretation of the matrix. You are correct that elements 1 and 2 of the parameter vector refer to the water basis and elements 3 and 4 to the bone basis. The poor condition number is at least partially related to the fact that the water and bone elements have very different thicknesses, and condition number is sensitive to the relative scaling of columns of the matrix.

   As the reviewer points out, the variances depend highly on the true values (“signal strength”) of the material thicknesses. Because condition number depends on scaling of the columns (or rows) of the matrix, we don’t believe the condition number of the covariance matrix is a meaningful way of summarizing the noise properties of our estimator, primarily because it doesn’t account for the size of the signal. For this reason, we chose not to discuss it in the paper. We do, however, acknowledge the large correlations in our estimator are a potential weakness.

   We have added sentences to the results section interpreting the diagonal elements of the covariance matrix to make clear, for example, that element (1, 1) being 5.45$mm^{2}$ means our estimate of the standard deviation of the estimator for a thickness of 20mm of water (based on our estimate for the left side of the detector) was $\sqrt{5.45}=2.33mm$. This puts the variances in context relative to signal strength. We additionally note the strong correlations as being a potential weakness of our approach in the discussion.

   Regarding the effect of bias on mean-squared error, we agree this is important, and needs to be addressed in future work, but we believe this is out of the scope of this proof-of-concept study. We have added a paragraph to the discussion acknowledging that the covariance matrix only characterizes the noise of our estimator and that bias was not investigated in this manuscript. The only claim we are trying to make in this manuscript is that, given enough dose (i.e., using as many photons as we need), this approach is feasible.
7) Comment:
   With the simulations already programmed, it should not be a large effort to vary the filtration in the “medical” case to see whether the information matrix might be equalized a bit to provide better estimation of the four components. Just working through the same type of approximate covariance structure would suffice. It is mentioned in the Discussion that filter optimization will be the subject of future work, but one perturbation would be nice to see here, too, and it could be fully explored in the next paper.

   Response:
   Thank you for this suggestion. We’ve done this, but would rather not complicate this present paper with such an analysis. The current paper already is demonstrating SPE with 2 different examples, and lays out a lot of different mathematical formalisms for realizing SPE with different energy ranges and detector types. We think it will start to get a little confusing to add optimization of any kind to this paper. We tried to pick simulation parameters that reflected a somewhat optimized set of parameters. So if one were to change the filter thickness by fractions of a mm, or the tissue thicknesses by a few cms, the results don’t change much as we presented here. If we saw much more drastic dependence on filter thicknesses and object thickness, we probably would try to get this behavior into the paper, as it would imply our methods are quite limited to specific problem instances. But we didn’t see that in our limited optimization work thus far.
